# Solvation of Na^+^, K^+^, and Their Dimers in Helium

**DOI:** 10.1002/chem.201103432

**Published:** 2012-02-28

**Authors:** Lukas An der Lan, Peter Bartl, Christian Leidlmair, Roland Jochum, Stephan Denifl, Olof Echt, Paul Scheier

**Affiliations:** [a] Institut für Ionenphysik und Angewandte Physik, Universität InnsbruckTechnikerstraße 25, 6020 Innsbruck (Austria) Fax: (+43) 512-507-2932; [b] Department of Physics, University of New HampshireDurham, NH 03824 (USA)

**Keywords:** alkali metal, helium, low temperature physics, mass spectrometry, solvation

## Abstract

Helium atoms bind strongly to alkali cations which, when embedded in liquid helium, form so-called snowballs. Calculations suggest that helium atoms in the first solvation layer of these snowballs form rigid structures and that their number (*n*) is well defined, especially for the lighter alkalis. However, experiments have so far failed to accurately determine values of *n*. We present high-resolution mass spectra of Na^+^He_*n*_, K^+^He_*n*_, Na_2_^+^He_*n*_ and K_2_^+^He_*n*_, formed by electron ionization of doped helium droplets; the data allow for a critical comparison with several theoretical studies. For sodium and potassium monomers the spectra indicate that the value of *n* is slightly smaller than calculated. Na_2_^+^He_*n*_ displays two distinct anomalies at *n*=2 and *n*=6, in agreement with theory; dissociation energies derived from experiment closely track theoretical values. K_2_^+^He_*n*_ distributions are fairly featureless, which also agrees with predictions.

## Introduction

Helium is a surprisingly efficient solvent; it binds strongly to many atomic, diatomic, and polyatomic cations.[1] The interaction between a cation and the dipoles induced in the surrounding helium atoms is significant. It often leads to the formation of one or more solvation shells; helium atoms located in these shells are highly compressed and localized. This so-called snowball greatly reduces the mobility of ions in liquid helium. Early measurements in pure helium suggested that each helium ion drags with it approximately 40 helium atoms.[2] However, the mobility of impurity ions injected into liquid helium, that is, the size of the snowball, depends on the nature of the ion.[3] Alkali ions injected into pressurized superfluid helium may even induce crystallization, that is, a transition to the solid phase.[4] On the other hand, infrared spectra of molecular ions embedded in helium nanodroplets containing more than 1000 helium atoms reveal surprisingly small matrix shifts that are not well understood.[5]

Alkali ions have attracted particular attention because of their strong binding to helium. The potential energy curves feature deep, narrow minima. The potential well for Na^+^–He is 40 times deeper than that for He–He.[6] Furthermore, the closed electronic shell of alkali ions ensures an isotropic interaction with the solvent and avoids complications that arise from the repulsive exchange interaction with unpaired valence electrons. Heavy singly charged alkaline earth metal ions, for example, may form bubbles rather than snowballs,[7–10] akin to the formation of bubbles around electrons injected into bulk helium or helium droplets.[11]

The microscopic structures, energetics, and dynamics of alkali-ion–helium complexes have been explored in several theoretical studies;[6, 8–10, 12–15] complexes formed with alkali dimer ions have been investigated as well.[16] By and large these studies agree on the main features: the radial density distributions reveal a distinct first layer of helium of high density, especially for the lighter alkali ions. Helium atoms in this first shell are immobile at low temperature. Their geometries often display high symmetry, including tetrahedral, octahedral and icosahedral symmetry;[9, 14] *I*_h_ symmetry may even extend to the second and third solvation shell.[10] The radius of the first helium shell increases monotonically from Li^+^ to Cs^+^, and so does the number of atoms (*n*), in the first shell. Rossi et al. used a variational approach with shadow wave functions and obtained values of *n*=12, 15 and 17.5 for Na^+^, K^+^ and Cs^+^.[8] Paolini et al. employed ground-state path integral Monte Carlo calculations and found *n*=8.2 for Li^+^ and 12.0 for Na^+^.[9] Coccia et al., based on variational and diffusion Monte Carlo calculations, reported completion of the first shell at *n*=10 for Li^+^ and between 11 and 12 for Na^+^.[13, 14] Galli et al. obtained *n*=12.0, 15.1 and 18.0 for Na^+^, K^+^ and Cs^+^, respectively. However, these last values apply to complexes that contain more helium atoms than needed to fill the first solvation shell; *n* is found to decrease slightly for smaller complexes.[10] Furthermore, the calculated value of atoms in the first solvation shell depends critically on minor details of the interaction potential between helium and the ion.[17]

Experimentally, the number of solvent atoms in a solvation shell is sometimes deduced from thermochemical data measured in the gas phase at thermal equilibrium.[18] Alternatively, one may try to infer the closure of solvation shells from anomalies in the ion yield of X^+^He_*n*_ measured by mass spectrometry. Prominent anomalies have been observed at *n*=12 for X=Ar,[19] Kr, Kr_2_, Kr_3_[20] and Pb,[21] *n*=10, 12, 32, 44 for Ag,[22] and *n*=4, 8 for Mg.[22] It is difficult to deduce any systematics from these data except that *n*=12 is often observed, probably because an icosahedral arrangement of 12 solvent atoms around the solvated ion is a particularly favorable arrangement.

Halogen cations, which show anomalies at *n*=10.2, 11.6, 13.5 and 15.9 for F^+^, Cl^+^, Br^+^ and I^+^, respectively, are the only systems for which experiments have established a correlation between *n* and the ionic size (the experimental values are non-integer because the ion yield did not change abruptly; *n* was obtained by fitting a smeared-out step function).[23] For alkali ions one expects a similar correlation between *n* and the ionic size. However, previous attempts to produce complexes of Na^+^ and K^+^ with helium were limited to very small sizes.[24] Mass spectra of helium droplets doped with Rb and Cs extended to larger sizes, but the observed anomalies were either caused by contaminants or smeared out over several cluster sizes.[24–26] As a result, a critical comparison of experiment and theory has not yet been possible.

In the present work we infer the presence of solvation shells for sodium and potassium ions and their dimers from high-resolution mass spectra, obtained by electron ionization of alkali-doped helium droplets that contain of the order of 5×10^5^ helium atoms. Strong fragmentation upon ionization leads to the formation of Na^+^He_*n*_, K^+^He_*n*_, Na_2_^+^He_*n*_ and K_2_^+^He_*n*_ ions that contain up to at least 20 helium atoms. Anomalies in the ion stability are deduced from anomalies in the ion yield by applying the evaporative ensemble.[27–29] Na^+^He_*n*_ and K^+^He_*n*_ show distinct anomalies that agree reasonably well with predicted shell closures although the experimental data suggest that the number of atoms in the first solvation shell is slightly overestimated by theory. For sodium dimer ions the agreement with theory[16] is excellent; dissociation energies deduced from mass spectra closely agree with theoretical values.

## Results

The most prominent ion series in mass spectra of helium droplets doped with sodium are Na_*m*_^+^, He_*n*_^+^, Na^+^He_*n*_, and Na_2_^+^He_*n*_; a weak series of Na_3_^+^He_*n*_ is observed as well. Sodium is monoisotopic (with a mass of 22.990 u), and He (mass 4.002603 u) is essentially monoisotopic; therefore Na^+^He_*n*_ and Na_2_^+^He_*n*_ ion peaks are well separated from each other, and also from He_*n*_^+^ and Na_3_^+^He_*n*_ ion peaks.

**Figure 1:**
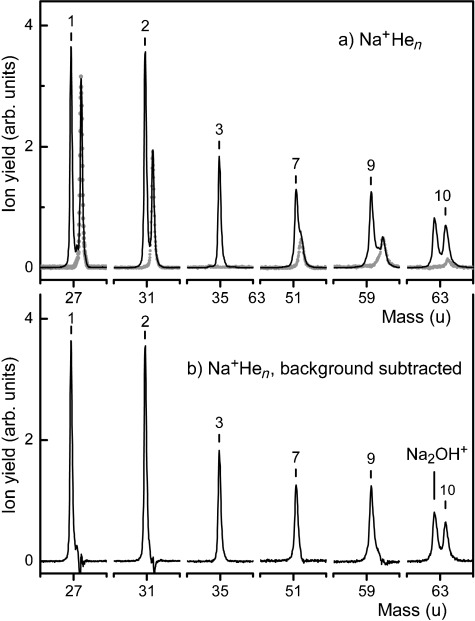
a) Solid lines: Six sections of a mass spectrum of helium droplets doped with sodium. All sections share the same linear *y*-scale. Each section covers a mass range of 0.2 u. Mass peaks assigned to Na^+^He_*n*_ are labeled by the value of *n*. Light grey (magenta online) dots represent a background spectrum, measured with undoped helium droplets. b) The mass spectrum of doped droplets after subtracting the background spectrum.

Sections of a mass spectrum are displayed in Figure 1 a by the solid line. Each section covers a mass range of 0.2 u; ticks are spaced at 0.1 u. The graph shows Na^+^He_*n*_ ions for *n*=1, 2, 3, 7, 9, 10; the sizes were chosen to demonstrate abrupt drops in the ion yield that occur at *n*=2 and 9. Na^+^He_*n*_ ion peaks are labeled by the value of *n*. Several additional ion peaks appear in the spectrum; the slight (≪1 u) shift to higher masses suggests that they are mostly due to hydrocarbons. For example, to the right of Na^+^He (mass 26.992 u) one observes another peak at 27.023 u, consistent with a C_2_H_3_^+^ contamination. Indeed, a background spectrum (full dots in Figure 1 a), recorded with the helium droplet beam on but the sodium source turned off, shows the same impurity peak at 27.023 u.

Subtracting the background spectrum from the spectrum of Na-doped helium droplets one obtains the spectrum in Figure 1 b. It shows only one peak other than Na^+^He_*n*_, namely to the left of Na^+^He_10_. Based on its mass of 62.983 u it is assigned to Na_2_OH^+^. The heavier analog of this hypermetallic ion, K_2_OH^+^, has been observed in a flow reactor study.[30] An ab-initio study shows that these monohydroxide ions have a planar, Y-shaped equilibrium structure with mostly ionic bonding.[31]

**Figure 2:**
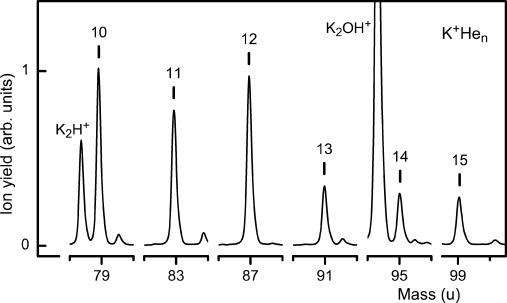
Six sections of a mass spectrum of helium droplets doped with potassium. All sections share the same linear *y*-scale. Each section covers a mass range of 0.2 u. Mass peaks assigned to ^39^K^+^He_*n*_ are labeled by the value of *n*.

Sections of a mass spectrum of helium droplets doped with potassium are shown in Figure 2; they show the presence of K^+^He_*n*_ for 10≤*n*≤15. Potassium has two isotopes; the ions in Figure 2 involve ^39^K (mass 38.9637 u, natural abundance 93.3 %). Two prominent ions other than ^39^K^+^He_*n*_ are seen as well. Based on their mass they are assigned to ^39^K_2_H^+^ and ^39^K_2_OH^+^. These ions do not, of course, appear in the background spectrum, which, quite generally, is void of intense ion peaks that could interfere with ^39^K^+^He_*n*_.

**Figure 3:**
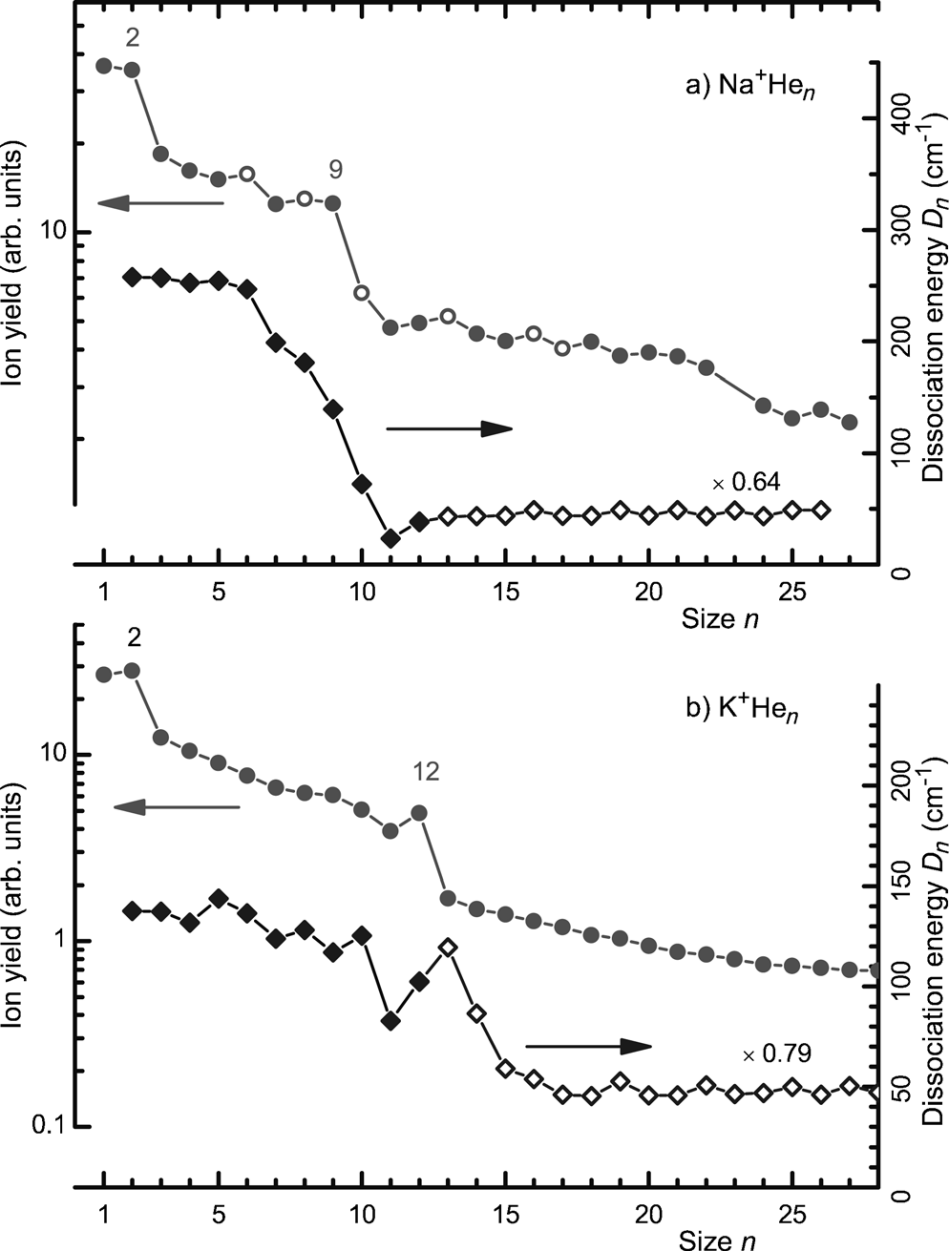
a) Size dependence of the experimental Na^+^He_*n*_ yield (full dots, left ordinate, logarithmic scale) together with dissociation energies calculated for *n* ≤12[14] (full diamonds, right ordinate, linear scale). Experimental values that suffer from interference with other mass peaks or poor statistics are represented by open dots.[32] Theoretical values for *n*>12 (open diamonds) are from Marinetti et al.;[15] they are scaled to match the *n*=12 value.[14] b) Similar to a) for K^+^He_*n*_.

The yield of Na^+^He_*n*_ and ^39^K^+^He_*n*_ extracted from mass spectra is displayed in Figure 3 a and 3b, respectively, on a semilogarithmic scale (left ordinate). Ion peaks that show no sign of significant contamination are represented by full dots; the estimated uncertainty is better than 10 %, about the size of the symbols. More problematic ion peaks,[32] such as Na^+^He_10_ (Figure 1 a), are shown as open dots; their uncertainties are probably below 20 %. No data point is shown for Na^+^He_23_^+^, which has the same nominal mass as the very intense Na_5_^+^ ion.

The Na^+^He_*n*_ series reveals two statistically significant intensity anomalies, namely abrupt drops by more than a factor two at *n*=2 and 9. ^39^K^+^He_*n*_ ions exhibit an abrupt drop at *n*=2 and a local maximum at *n*=12; these anomalies have been confirmed by analyzing the yield of ^41^K^+^He_*n*_.

**Figure 4:**
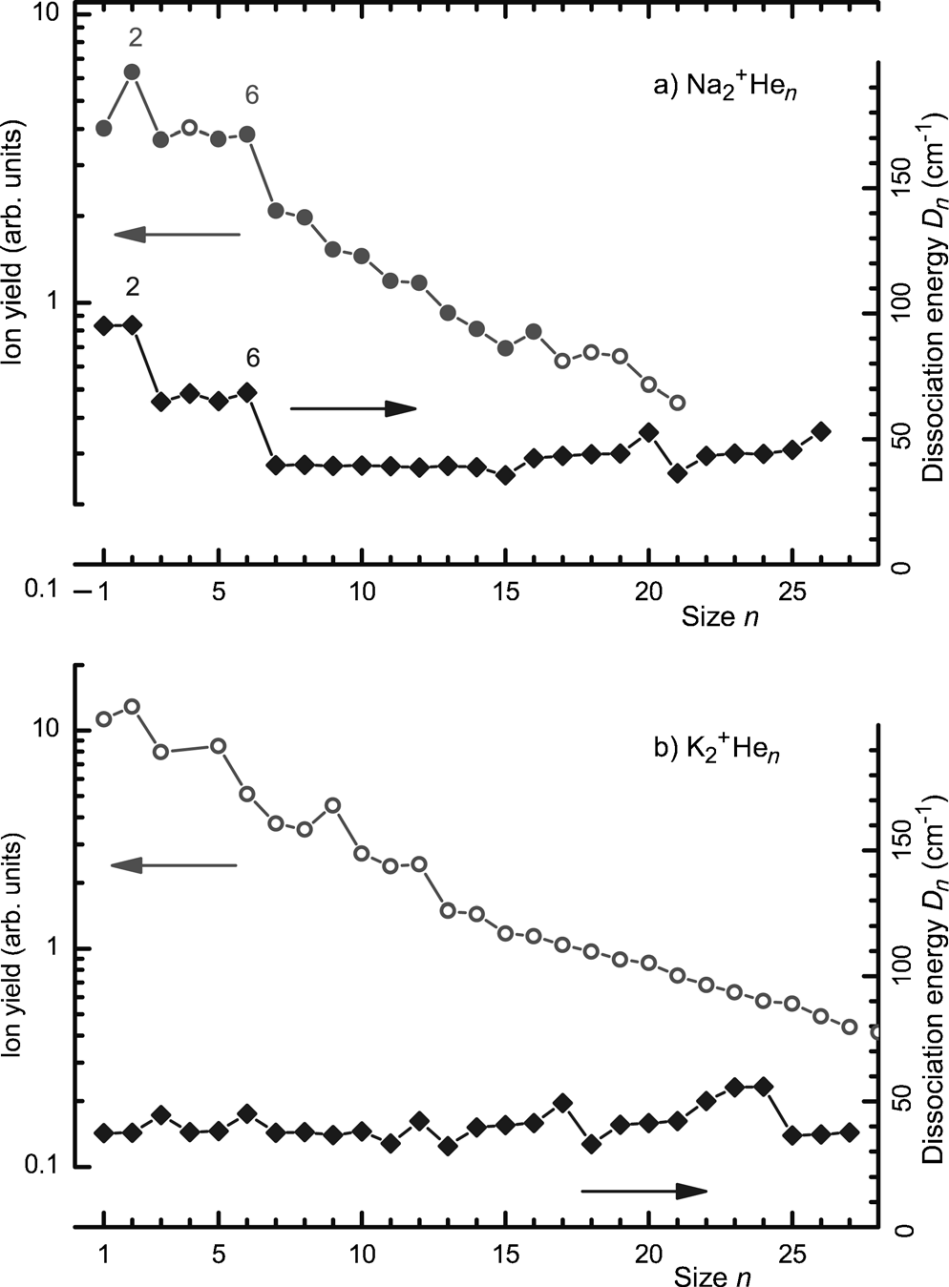
a) Size dependence of the experimental Na_2_^+^He_*n*_ yield (filled symbols, left ordinate, logarithmic scale) together with calculated[16] dissociation energies (right ordinate). Experimental values that suffer from interference with other mass peaks or poor statistics are represented by open symbols. b) Similar to a) for K_2_^+^He_*n*_.

The yields of sodium and potassium dimer-helium complexes are displayed in Figure 4 a and b, respectively. Na_2_^+^He_*n*_ shows a local maximum at *n*=2 and an abrupt drop at *n*=6. Several minor anomalies are seen in the ^39^K_2_^+^He_*n*_ series, but an analysis of the ^39^K^41^K^+^He_*n*_ series reveals that their statistical significance is questionable. The data point for ^39^K_2_^+^He_4_ has been omitted, because the ion is swamped by a strong K_2_O^+^ ion peak; oxides are a common nuisance in studies of alkali clusters.[33]

## Discussion

An intense, highly stable helium droplet source combined with a high-resolution mass spectrometer has made it possible to unambiguously identify alkali-helium complexes M_*m*_^+^He_*n*_ in which M=Na or K, *m*=1 or 2, and *n* extends to 20 or larger. With the exception of K_2_^+^He_*n*_, the ion series exhibit distinct anomalies in the ion yield versus size *n*. These anomalies were not observed in a previous study of alkali–helium complexes by Stienkemeier and co-workers, primarily because their ion yields decreased too quickly with increasing *n*; no complexes were observed for *n*>8.[34]

In principle, anomalies in the ion yield *I_n_* of clusters may be caused by several factors including kinetics, size-selective ionization, or anomalies in the microcanonical heat capacities *C_n_*. For atomic clusters that are prone to fragmentation upon ionization the most likely cause are anomalies in the dissociation energies *D_n_* (often called evaporation energies), that is, the difference between total energies *E_n_* of cluster ions of adjacent size in their most stable configurations [Eq. (1)]


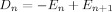
(1)

The relation between the size dependence of *D_n_* and *I_n_* has been explored by several authors,[28, 29, 35] based on the model of the evaporative ensemble.[27] Key ingredients of this model are that the initial cluster distribution is broad, dissociation is a statistical process, and each cluster ion that is observed has undergone at least one evaporation. The small heat capacity of clusters containing less than *n*≍10^2^ units ensures that each evaporation cools the cluster significantly, thus leading to a drastic (at least a factor 10) reduction of the rate coefficient *k*. An ensemble of cluster ions X_*n*_^+^ that continues to be populated by evaporation from X_*n*+1_^+^ and depopulated by evaporation into X_*n*−1_^+^ will, if investigated at time *t* after ionization, feature an average rate coefficient *k*≍1/*t*. Furthermore, the ensemble will be characterized by rather well-defined upper and lower limits to its (vibrational) excitation energy E_n_*.[36] The energy limits are related to the dissociation energies *D_n_* and *D*_*n*+1_, respectively. An upper limit exists because very hot X_*n*_^+^ would rapidly dissociate into X_*n*−1_^+^; a lower limit exists because very cold precursor ions X_*n*+1_^+^ will not dissociate into X_*n*_^+^ on the experimental time scale.

So far we have merely summarized basic ideas underlying the evaporative ensemble.[27] If, furthermore, each cluster has suffered multiple evaporations, that is, if the initial excitation energy E_n_* greatly exceeds *D_n_* and is broadly distributed, then the initial size distribution of cluster ions will be projected onto the final one;[29] any features in the size distribution of the neutral precursors will be wiped out by the statistical nature of dissociation. The distribution of excitation energies of X_*n*_^+^ will be approximately rectangular and, if the initial size distribution was very broad, the observed yield of X_*n*_^+^ versus size *n* will be proportional to the width of the energy distribution. With some approximations one can write Equation (2).[37]


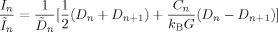
(2)

The microcanonical heat capacities *C_n_* appear because rate coefficients are expressed in terms of the microcanonical temperature *T*(E*); one often assumes that *C_n_* is given by the equipartition theorem, *C_n_*=(3*n*−7)*k*_B_. The Gspann factor *G* enters because the energy limits depend (logarithmically) on the timescale.[27, 38]

The quantities 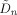 and 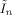 in Equation (2) are local averages of *D_n_* and *I_n_* over just a few cluster sizes around *n*. They may be obtained from local averages with Gaussian weighting,[37] or by fitting a smooth function, for example, a low-order polynomial. In the absence of anomalies, 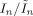 would equal 1.0 for all values of *n*; local anomalies in the experimental quantity 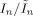 thus imply local anomalies in *D_n_*. The approach has been applied to derive, for example, relative dissociation energies of Ar_*n*_^+^ and Xe_*n*_^+^ from mass spectra.[37]

A special situation arises if *C_n_* is much less than the classical value; this applies to ions complexed with a few helium atoms. Here the interaction among atoms in the solvation shell is typically much weaker than the interaction with the solvated ion which carries no internal energy; even a large polyatomic ion such as C_60_^+^ will be cooled in helium to its vibrational ground state.[17] In this case Equation (2) is to be replaced by Equation (3).


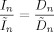
(3)

This relation has been used recently without a formal derivation;[17] it is readily understood by reconsidering the discussion that led to Equation (2). What happens if the system has zero heat capacity? In this case the upper energy limit becomes equal to *D_n_* because the lifetime of X_*n*_^+^ would be zero for higher energies and infinite for lower values. On the other hand, the lower energy limit becomes zero because, if a precursor X_*n*+1_^+^ has just enough energy to dissociate, *E*_*n*+1_*=*D*_*n*+1_, it will produce X_*n*_^+^ with E_n_*=0. Equation (3) follows from the notion that the yield *I_n_* is proportional to the width of the energy distribution, *D_n_*−0. Again, the smoothly varying (marked with a tilde) terms in Equation (3) enter because the constant of proportionality may slowly vary with *n*, for example because the initial neutral cluster size distribution has a finite width, detection efficiencies may depend on *n*, and so forth.

A more general analysis of systems that have very small but non-zero heat capacities indicates that Equation (3) provides a good approximation provided *C_n_*≤20 *k*_B_.[39] This result may be understood by the following consideration: The small heat capacity *C* that was assumed in the derivation of Equation (3) implies that evaporations will quickly reduce the thermal (vibrational) energy E* of the ions to values below their dissociation energy, that is, E*=∫*C*d*T*<*D*. Is that a reasonable assumption for the systems considered here? For a numerical example we consider Na_2_^+^He_*n*_ which exhibits an abrupt drop in ion yield between *n*=6 and 7. The corresponding theoretical dissociation energies are *D*_6_=68.5 and *D*_7_=39.6 cm^−1^, respectively.[16] The usual assumption that temperatures of small systems are proportional to their dissociation energies[40] would imply temperatures of *T*_6_=5.1 and *T*_7_=2.9 K. To estimate the heat capacity of the cluster ions we consider submonolayers of helium adsorbed on graphite; this system also features strong interaction of helium with the substrate (Na_2_^+^ in our case) as opposed to weak interaction within the adsorbate. The experimental heat capacity of helium on graphite in the √3×√3 phase, above the commensurate–incommensurate phase transition at 3 K, is about 0.3 *k*_B_ per atom, or 10 % of the classical value.[41] By ignoring the decrease in *C* with decreasing temperature mandated by the third law of thermodynamics we thus obtain an upper limit for the thermal energy of the ions, E_n_*<*C_n_T_n_*≍0.1 *D_n_*. The result shows that our assumptions are justified although a more rigorous analysis that avoids the concept of temperature altogether would be desirable. Note that the evaporative model in its usual form, that is Equation (2), will not apply to systems with very small heat capacity; there is no such inherent limit to Equation (3).

We emphasize that the present work concerns solvated alkali ions; the results are not affected by the unusual properties of their neutral precursors: Whereas alkali ions bind strongly to helium, neutral alkalis interact very weakly. Helium barely wets extended surfaces of sodium or potassium.[42] Small sodium and potassium clusters remain on the surface of helium droplets;[43] they do not submerge unless they contain at least ≍20 atoms.[44, 45] Thus, a dramatic rearrangement of the solvent will happen upon ionization. Some interesting details about the dynamics following photoionization have been published,[46] but they are unlikely to apply to electron ionization which involves Penning ionization, that is, formation of an intermediate, electronically excited helium atom with subsequent energy transfer to the alkali.[47] The ionization dynamics may also differ considerably from those following electron ionization of submerged dopants which involves charge transfer from an intermediate He^+^.[48] Electron ionization of doped helium droplets always implies large excess energies (with ionization thresholds at or above 19.8 eV) and strong fragmentation, including fragmentation of alkali cluster ions.[45, 49] A likely scenario is ejection of the nascent alkali (cluster) ion complexed with some helium and subsequent evaporation until the average rate coefficient is reduced to 1/*t*. At any rate, the large amount of excess energy that is available guarantees strong fragmentation which in turn guarantees that any features in the neutral distribution are wiped out, that is, a crucial assumption made in the derivation of Equation (3) is met.

We rewrite Equation (3) to deduce experimental dissociation energies from the measured ion yield [Eq. (4), in which 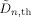 is the local average of theoretical dissociation energies *D*_*n*,th_].


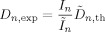
(4)

Several groups have computed the microscopic structures of cationic-metal–helium complexes;[6, 8–10, 12–15, 50] in some cases total energies *E_n_* of the complexes were calculated as well. However, dissociation energies *D_n_* can only be deduced if *E_n_* is computed for a continuous range of *n* values; the only such studies have been reported by Slavicek and Lewerenz for Pb^+^ and Pb^2+^,[50] and by Gianturco and co-workers for Li^+^, Na^+^, K^+[14]^ and Li_2_^+^, Na_2_^+^, K_2_^+^.[16] Dissociation energies computed for sodium and potassium monomers are displayed in Figure 3 (solid diamonds, right ordinate). The data[14] were limited to *n*≤12; Figure 3 also includes dissociation energies from an earlier calculation,[15, 51] scaled to match the more recent data at M^+^He_12_. Dissociation energies computed for Na_2_^+^He_*n*_ and K_2_^+^He_*n*_[16] are plotted in Figure 4 (solid diamonds, right ordinate).

There are some similarities but also significant differences between experimental and theoretical data in Figure 3. Sodium and potassium both show a significant (factor 2) drop in the ion yield beyond M^+^He_2_ which is absent from the calculated dissociation energies. In principle, experimental ion yields represent only upper bounds; contaminants may remain undetected. However, we do not observe any background near the mass of M^+^He_2_. The only possible interference that we can think of would be (M_2_O)^2+^. This dication, if it were to survive charge separation, would have a mass-to-charge ratio 0.008 u e^−1^ below that of M^+^He_2_. This difference is approximately equal to the width (FWHM, full-width-at-half-maximum) of ion peaks in this mass range; therefore a (M_2_O)^2+^ contamination can be excluded.

Experimental dissociation energies of Na^+^He_*n*_ show another abrupt drop at *n*=9, while calculated dissociation energies decrease gradually between 6 and 11. An analysis of the calculated radial distribution functions reveals that the first solvation shell is probably completed when *n* reaches 11 or 12,[14] in good agreement with calculations by other researchers.[8, 9] However, Galli et al. who employed a path-integral Monte Carlo method report a value of 10. The exact number of atoms in the first shell actually depends on the total number of helium atoms in the complex;[10] it tends to increase with increasing droplet size because the density of helium in the core increases as well. The value of 10 refers to droplets containing ≤30 helium atoms at a temperature of 1 K whereas the value 9 in our experimental data refers to a complex that contains exactly nine helium atoms. On the other hand, its temperature is probably higher than 1 K (to the extent that one may speak of a temperature) because the temperatures of evaporating clusters scale with their dissociation energies;[40] the systems considered here are at least an order of magnitude more strongly bound than neutral helium droplets (dissociation energy 5 cm^−1^) which cool to ≍0.37 K.[52]

The gradual decline in dissociation energies calculated for K^+^He_*n*_ up to *n*=10 agrees with experimental data, except for the drop at K^+^He_2_. The ion yield exhibits a distinct local maximum at *n*=12. Calculated dissociation energies (full diamonds) follow the same pattern up to *n*=12 which was the largest size in that study.[14] The earlier theoretical values (open diamonds[15, 51]) show an increase from *n*=12 to 13 which does not correlate with the experimental ion yield.

The radial distribution function computed for K^+^He_*n*_ by Rossi et al.[8] indicates that the first solvation shell fills at *n*=15. However, Galli et al.[10] reported that K^+^He_14_ has one helium atom outside the first solvation shell, that is, *n*=13. Thus, the anomalies observed in the experiment (at Na^+^He_9_ and K^+^He_12_) are just one unit less than the number of helium atoms computed by Galli et al.[10] for the first solvation shell in small droplets; the disagreement is slightly larger if our data are compared with other[8, 9, 14] theoretical studies.

**Figure 5:**
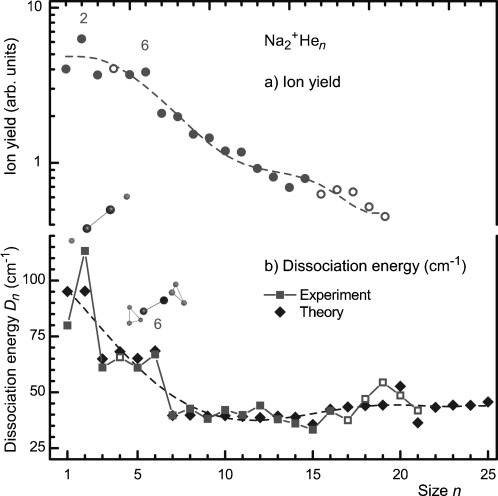
a) Experimental ion yield *I_n_* of Na_2_^+^He_*n*_ together with a fitted smooth line representing 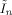 [see Eq. (3)]. b) Calculated dissociation energies *D*_*n*,th_ (diamonds[16]) together with a fitted smooth line representing 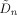. Squares: experimental dissociation energy *D*_*n*,exptl_ computed from *I_n_* with help of Equation (4).

The data for Na_2_^+^He_*n*_ (Figure 4 a) show striking agreement between experiment and theory,[16] namely abrupt drops at *n*=2 and 6. We apply Equation (4) to deduce experimental dissociation energies *D*_*n*,exptl_ from the measured ion yield. *I_n_* is displayed in Figure 5 a together with the smooth function 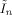 obtained by fitting a polynomial of 5^th^ order to *I_n_* (dashed line). The order was chosen somewhat arbitrarily; the exact value does not matter. The panel in Figure 5b shows the calculated[16] dissociation energies *D*_*n*,th_ (diamonds) from which the smooth function 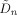 is obtained by a 5th order polynomial fit (dashed line). Experimental dissociation energies *D*_*n*,exptl_ are shown as squares connected by solid lines. *D*_*n*,exptl_ closely tracks *D*_*n*,th_ from *n*=3 to 11; reduced experimental accuracy prevents a critical comparison for much larger values of *n*. The magnitude in the abrupt drop at *n*=6 is the same for both data sets, about 40 %. We reiterate that the use of averaged (marked with a tilde) functions in Equation (4) guarantees that the overall shape and absolute values of *D*_*n*,exptl_ equal those of *D*_*n*,th_, but the quantitative agreement in the local anomalies is a significant, non-trivial result.

Also indicated in Figure 5 b are the ground state structures calculated for *n*=2 and 6.[16] Na_2_^+^He_2_ is linear with the two He atoms positioned at opposite ends of the alkali dimer. The first shell closes when each alkali atom is capped by three He atoms in a rigid arrangement; further helium atoms are then added independently to these two caps in a more delocalized fashion.[16]

The interaction of He with K_2_^+^ is much weaker than with Na_2_^+^; therefore the localization of He atoms in K_2_^+^He_*n*_ is less pronounced.[16] The calculated dissociation energies of K_2_^+^He_*n*_ do not show any anomalies in the dissociation energies upon completion of subshells even though the classical structures are comparable to those of Na_2_^+^He_*n*_. The experimental data (Figure 4 b) show minor anomalies at *n*=2, 5, 9 and 12, but their statistical significance is borderline. As a further check we have analyzed the ^39^K^41^K^+^He_*n*_ series, which is an order of magnitude weaker than the ^39^K_2_^+^He_*n*_ series; the data suggest that potassium-dimer–helium complexes feature no statistically significant anomalies.

Cationic alkali–helium complexes have been studied by Stienkemeier and co-workers[34] and Ernst and co-workers.[25, 26] Müller et al. employed multiphoton ionization of doped helium droplets with a femtosecond laser.[34] The maximum number of helium atoms attached to Na^+^, Na_2_^+^ or K^+^ was 8 or less; the distributions were void of any significant anomalies. The Rb^+^He_*n*_ distribution extended to *n*=35 with several gaps in between due to interference with contaminants; it revealed no prominent anomalies. For Cs^+^He_*n*_ a gradual change in slope was noticed between *n*=15 and 20; calculations predict completion of the first solvation shell in this range.[8, 10] Theisen et al.[26] ionized Cs-doped droplets with laser pulses of 30 ns duration at a photon energy of 2.74 eV, well below the ionization energy of the Cs atom. Their spectra confirm the gradual change of slope between *n*=15 and 20. They also determined the ion yield of Cs_2_^+^He_*n*_ for *n*≤23, which showed two pronounced local maxima at *n*=4 and 8. The authors tentatively assigned the first anomaly to an artifact arising from a strong Cs_2_OH^+^ signal, but did not explain the anomaly at *n*=8 which was not observed by Müller et al.[24] The anomaly may be due to Cs_2_O_2_^+^. Cesium readily forms highly oxidized clusters,[53] and the mass difference of 0.0155 u between Cs_2_O_2_^+^ and Cs_2_^+^He_8_ would have been well below the resolution limit.

At any rate, the binding of helium to Cs^+^ or Rb^+^ is much weaker than for the lighter alkalis. Therefore the He–He interaction plays a relatively large role in Rb^+^He_*n*_ and Cs^+^He_*n*_; this tends to blur the distinction between the first and second solvation shell and makes anomalies in the size dependence of dissociation energies less distinct.[10, 14] Sodium ions are much better suited for a critical comparison of theoretical and experimental data than complexes involving rubidium or cesium.

As a final note we point out a possible trend. Features in Na^+^He_*n*_ and K^+^He_*n*_ distributions are observed at *n*=9 and 12, slightly below the values where the first solvation shell closes according to calculated radial distribution functions;[6, 8–10, 14] values computed by Galli et al.[10] are just one unit higher than the experimental values. Similarly, distributions of C_60_^+^He_*n*_ measured by our group[17] show an anomaly in the ion yield at *n*=60, whereas the calculated number of helium atoms in the first solvation shell is somewhat larger. The exact value of *n* turns out to be very sensitive to details of the interaction potential between helium and C_60_^+^.[17] It is tempting to speculate that the disagreement between experimental and theoretical value found in the present work is also due to minute details of the interaction potential, although differences in temperature may also play a role.

## Conclusion

We have compared experimental ion yields of alkali monomer and dimer ions complexed with helium with calculated dissociation energies. These two quantities should closely correlate if the heat capacities of the cluster ions are small compared to the classical equipartition values. Several distinct anomalies have been identified in the ion yields that agree closely (for M^+^He_*n*_) or even exactly (for Na_2_^+^He_*n*_) with computed dissociation energies, but there are also significant differences. It would be interesting to perform similar experiments with lithium. Li^+^ and Li_2_^+^ bind much more strongly to helium than the heavier alkali ions. Anomalies in calculated dissociation energies are more pronounced, on an absolute as well as a relative scale;[14, 16] corresponding anomalies in the ion yield should therefore be more prominent as well.

## Experimental Section

Neutral helium nanodroplets were produced by expanding helium (purity 99.9999 %) from a stagnation pressure of approximately 2mPa through a 5 μm nozzle, cooled to about 8 K, into vacuum. The average number of atoms per droplet formed in the expansion is of the order of 5×10^5^; the droplets are superfluid with a temperature of ≍0.37 K.[52] The resulting supersonic beam was skimmed by a 0.8 mm conical skimmer, located 8 mm downstream from the nozzle. The skimmed beam traversed a 20 cm-long pick-up region into which metallic sodium or potassium (Sigma Aldrich, purity 99.95 %) was vaporized from a crucible kept at 270 and 100 °C, respectively. Conditions were tuned to favor the pickup of one or just a few alkali atoms per droplet.

After the pick-up region the doped helium droplets passed a region in which they were ionized by electron impact at energies of 60 or 70 eV. No significant effect of the electron energy on the mass spectra was observed; data shown in this work were recorded at 70 eV.

Cations were accelerated to 40 eV into the extraction region of a commercial time-of-flight mass spectrometer equipped with a reflectron (Tofwerk AG, model HTOF); its mass resolution is about Δ*m*/*m*=1:5000. The base pressure in the mass spectrometer was 10^−5^ Pa. The ions were extracted at 90° into the field-free region of the spectrometer by a pulsed extraction voltage. At the end of the field-free region they entered a two-stage reflectron which reflects them towards a microchannel plate detector operated in single ion counting mode. Additional experimental details have been described elsewhere.[45, 54]
